# The Long-Term Dynamics of the Particulate ^137^Cs Supply from Eroded Arable Slopes During the Post-Chernobyl Period

**DOI:** 10.3390/toxics14040344

**Published:** 2026-04-19

**Authors:** Maksim M. Ivanov, Polina Fominykh, Nadezhda Ivanova, Sergei Krasnov, Valentin Golosov

**Affiliations:** 1 Faculty of Geography, Lomonosov Moscow State University, Moscow 119991, Russia; foma41polina@gmail.com (P.F.); nadine_iv@mail.ru (N.I.); skras@mail.ru (S.K.); gollossov@gmail.com (V.G.); 2 Institute of Geography, Russian Academy of Sciences (RAS), Moscow 119017, Russia

**Keywords:** Chernobyl contamination, erosion, acceleration erosion, sediment load, radionuclide migration

## Abstract

In rural areas affected by Chernobyl, accelerated erosion has become a major source of particulate ^137^Cs in sediment load. The long-term dynamics of the activity concentration in eroded soil material transported from individual slope catchments can be better understood by exploring the ^137^Cs depth distribution in sediments deposited near cultivated fields. This study focuses on three cultivated slope catchments located in the Chernobyl-affected area of Central Russia. A depth incremental campaign was conducted within zones of sediment accumulation in 2022–2025. The behavior of radiocaesium associated with particles after the Chernobyl accident was controlled by the prompt implementation of remediation measures. Shortly after the accident, the values decreased by more than two times. The radionuclide flux then began to depend on soil erosion processes. Gradually, the thickness of the upper soil that had been eroded became large enough to allow soil material from deeper layers to be involved during ordinary plowing and led to a subsequent decrease in the ^137^Cs activity concentration. Given the decreasing snowmelt runoff and lack of increase in high-intensity rainfall in the 21st century, the activity concentration of ^137^Cs in slope runoff has remained quite stable. This phenomenon requires consideration of whether a physically based model for the transport of particulate radionuclides should be developed.

## 1. Introduction

Accelerated soil erosion is considered one of the major human impacts on the environment. The displacement and transportation of soil from arable slopes without natural canopy cover leads to land degradation worldwide [1,2,3,4,5]. In addition to the depletion of soil resources, land erosion acts as a source of sediment and particle-bound chemical compounds for riverine systems, which, in turn, leads to the deterioration of water resources [6,7,8].

Radioactive contamination of river systems has a special place among other environmental issues. On the one hand, contamination is a major challenge in areas affected by intense fallout of technogenic radionuclides, such as those in Chernobyl and Fukushima. Its solution requires both immediate emergency measures directly after fallout [9,10] and long-term strategies for remediation decades later [11,12,13,14]. On the other hand, artificial radionuclides, especially ^137^Cs, are commonly used as tracers in erosion and sedimentation studies [15,16,17,18]. In addition, the study of anthropogenic radionuclide fallout can aid in understanding the long-term fate of particle-bound contaminants in river basins and can provide a basis for planning remedial measures or exploiting natural attenuation processes.

Studying the depth distribution of radionuclides in sediment sinks can aid in identifying changes in mobilized and subsequently deposited sediment loads [19,20,21,22]. This information is highly valuable in the absence of direct observation, even when regular monitoring techniques are used. However, records in sedimentary archives should be studied with great caution, considering the limitations imposed by the irregular nature of sediment transport and accumulation and possible disturbances after deposition.

The purpose of this work is to elucidate the long-term changes in the ^137^Cs activity concentration of sediments delivered from arable slopes in the Chernobyl-affected area in the Central Russian Upland, which can be determined by examining sediments accumulated in proximity to arable slopes. First, three small agricultural catchments were selected, where depth incremental sampling campaigns were conducted between 2022 and 2025 along the expected sediment transport path. Second, the representativeness of the obtained results was analyzed based on the transportation distance and the geomorphic conditions of the accumulation area. As water erosion is the main process for sediment mobilization and transportation, as well as natural attenuation, areas that could contribute to the sediment load were identified, and the mean erosion rates during the post-accident period were estimated via mathematical models. The depth distribution of ^137^Cs was subsequently studied and analyzed at the sampling points, considering the intensity of erosion conditions in the sedimentation process.

## 2. Materials and Methods

**Figure 1:**
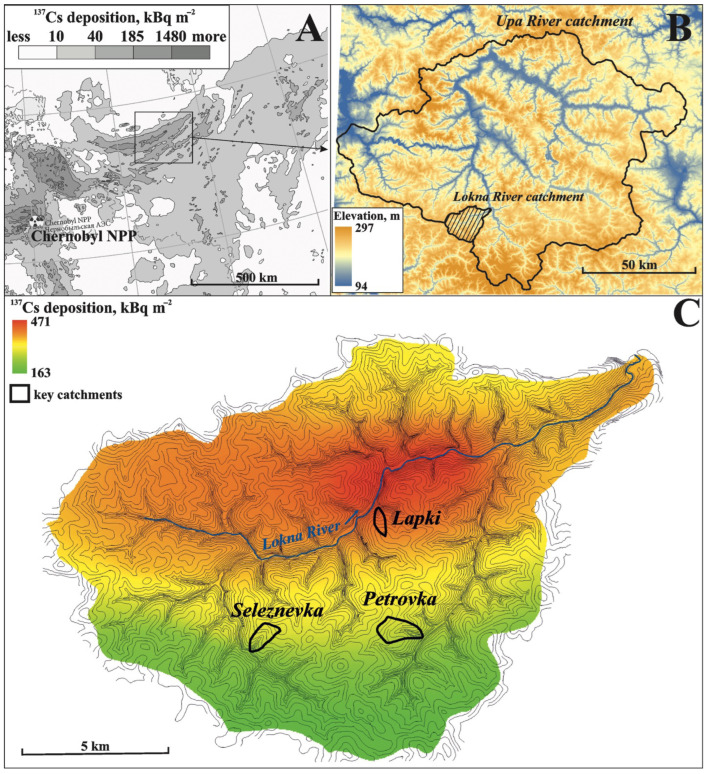
Location of the Upa River basin on the map of the Chernobyl fallout [27] (**A**); digital elevation model (FABDEM) of the Upa River basin (**B**); and location of key catchments in the Lokna River basin (**C**).

The study sites are located in the highly contaminated area of Central Russia, which was known as the “Plavsk hot spot” after the Chernobyl accident (Figure 1A). The “hot spot” is a relatively narrow strip that has a sublatitudinal shape and stretches for more than 100 km, mostly occupying the area of the Upa River basin. The latter covers an area of approximately 9500 square kilometers in the northern part of the Central Russian Upland in the Tula region (see Figure 1B). The bedrock of the region is primarily composed of limestone from the Carboniferous period, which is overlain by a layer of loess-like loam that serves as a parent rock for the soils. According to the World Reference Base for Soil Resources (WRB-2022), the soils in the interfluves are represented by Luvic Chernic Phaeozems, Luvic Greyzemic Chernic Phaeozems, and Dark-Gray Forest Soils. A previous investigation showed that the soil cover has a homogeneous mechanical composition in areas where the A horizon has not been eroded to bedrock. More than 50% of the particles are in the size range of 8–63 μm. The maximum particle size does not usually exceed 125 μm. Overgrain size fractions are minor [23].

The region is located in the Dfb climate zone according to the Köppen–Geiger classification, with a cold climate with warm summers and no dry season [24]. The annual precipitation varies from approximately 630 mm in the northwest to 592 mm in the southeast, with an average rainfall of approximately 469 mm during the warm season. According to data from the Plavsk meteorological station, precipitation can vary by more than two times from year to year. More than half of the rainfall occurs on the 12 days with the highest precipitation. Observations in the forest-steppe zone indicate that spring runoff varies widely from year to year, and a trend toward a steady decrease in runoff can be seen between 1959 and 1996. However, this trend has not been observed in the last 20 years, likely because of changes in hydrometeorological conditions and other natural factors affecting runoff. In recent decades, there has been a significant increase in snowmelt absorption, averaging at 25–45% [25]. These climatic conditions likely lead to a reduction in erosion during spring snowmelt and a lack of significant growth during warmer months.

Three small key catchments were explored in the most contaminated area, namely, the Lokna River basin (see Figure 1C). All of these catchments have been studied as part of the more than 30-year history of radiocaesium studies on the erosion and migration of radionuclides in the Upa River basin [26]. This study consists of three components: field geomorphic investigations, radiocaesium depth incremental studies, and erosion modeling.

The field geomorphic study was conducted with two main objectives. The first was to determine the actual area of arable slopes that could contribute to sediment loads entering the river system. According to Panin et al. [28] and later clarified by Ivanov et al. [29], eroded material has a limited chance of overcoming the lower boundary of cultivated fields and being transported further along the river network. Constant mechanical impact on the soil during cultivation leads to a transformation of the longitudinal profile of the slope parallel to the main direction of plowing [30]. This phenomenon is common in agricultural landscapes around the world, where the formation of agricultural terraces and benches is a result of the combined effects of moldboard plowing and the accumulation of sediments mobilized on arable land [31,32,33]. The most common terms for this agricultural landform in English are “lynchet” and “Celtic field”. As reported by Koshovskii et al. [34], the lower boundary of long-cultivated fields is separated from valley slopes by ridges that are up to several tens of centimeters in height. This height is sufficient to block runoff from the slope and trap sediments. The morphology of the lynchet changes due to sediment accumulation, and the possibility of overflow increases. Overflow may occur if the concentration of runoff at the foot of the slope is high or if the slope has previously eroded. Such situations occur mainly in the lower parts of slopes or in the corners of fields, where water flows down from higher elevations along the lynchets. Field geomorphic observations are essential because dense vegetation cover makes it impossible to fully assess the conditions of the lower boundary of arable slopes via satellite imagery or even UAV photography.

The second objective was to explore sediment transport pathways in order to identify representative sampling points. Sediment accumulation is a process with high spatial variability. Its intensity can vary greatly, even within very close areas, depending on topographical features. Thus, the geomorphological positions of the sampling points largely determine the scope of interpretation of the data obtained. When selecting a position, we began with the assumption that sampling would be most effective if the flow concentration was at a maximum and that there would be no obvious indications of disturbances to the sedimentation process. We used the following as the main geomorphic criteria for the sampling location: the lowest possible location on a chosen cross-section (thalweg), the absence of fresh erosion incisions, and any significant changes in morphological parameters (slope angle, valley bottom width, or local sediment sink). Further speculation on this topic and related limitations in interpreting radiocaesium data can be found in Section 4.

### 2.1. Radiocaesium Depth Increment Study

Prior to the in-depth study of the ^137^Cs distribution, each accumulation site was thoroughly examined. The selection of sampling sites was not arbitrary but rather based on a thorough analysis of geomorphological characteristics. The sites were selected based on the anticipated sediment transport and deposition rates at these locations. In particular, this was related to variations in surface roughness (cultivated fields and vegetated slopes) and slope inclination, as well as changes in the width of the valley bottom. It was then decided to collect sediment samples from the walls of the soil sections.

Before sampling, we developed a detailed description of the soil profile. This approach is labor-intensive, but it allowed us to detect and prevent disturbances in the ^137^Cs depth distribution caused by the soil bioturbation process. These disturbances can be caused by animal or human activities or plant roots. Any additional evidence, such as soil texture, which indicates steady sedimentation or disturbance of the soil, was recorded. Therefore, this approach allowed us to focus on single yet representative points, rather than averaging samples taken randomly. Each sample that we collected had the shape of a parallelepiped with well-defined dimensions, allowing for calculation of the radionuclide inventory and construction of the vertical distribution of radionuclides. The sampling interval was 3 cm, and the horizontal cross-sectional area of the samples was 10 × 10 cm. The maximum sampling depth varied from point to point, depending on the observed thickness of the accumulated strata. To avoid underestimating the necessary sampling depth, we used a portable dosimeter. If the dose rate was lower at the bottom of a soil section and a maximum was detected in the middle, the depth was considered sufficient.

Exceptions were made for two points in the Lapki catchment (LF-3 and LS-3 points). At these points, samples were collected via a hand auger with a diameter of 3 cm and were sliced into 3 cm thick sections in the field. The sampling device did not allow us to accurately determine the size of the horizontal cross-sectional area, and a precise assessment of the ^137^Cs inventory was therefore impossible. Nevertheless, the depth distribution of the activity concentration was considered relevant, as it was verified by duplicating the sediment core at the same location.

The collected samples were taken to the laboratory, where they were weighed before and after drying to determine their mass and moisture content. Each sample was then ground to a size of up to 2 mm and placed in a 50 mL cylinder (Sarstedt multi-purpose container Lx∅: 55 × 44 mm) prior to analysis of ^137^Cs via gamma spectrometry with an ORTEC coaxial HGe detector (GEM30P40) (AMETEK ORTEC, Oak Ridre, TN, USA). To calculate the photopeak efficiency, certified reference material IAEA-447 (International Atomic Energy Agency, Vienna, Austria) was used. The exposure time for the samples was chosen to ensure a precision of at least ± 7%. All activity values were then decay-corrected to 1986. The sampling procedure was conducted over a period of several years, from 2022 to 2025. Given the reduced snowmelt runoff and expected moderate erosion rates [25] and sediment supply, we assumed that a 4-year gap was too short to observe meaningful changes in the depth distribution of ^137^Cs. Therefore, it was assumed that all the data obtained corresponded to the same time frame after the Chernobyl incident, both within and between the studied key catchments. Even if a single intense erosion event occurred between the sampling campaigns, its impact is likely to be minor compared to that of the entire period under study and would not significantly affect the overall picture of post-Chernobyl sedimentation.

### 2.2. Erosion Modeling

Soil erosion losses were simulated using the SERSAL model (Soil Erosion from Rain and Snow on Agricultural Land). This is a two-dimensional model that best meets practical needs [35,36] and represents the well-known Universal Soil Loss Equation (Wischmeier–Smith equation, USLE) [37] implemented in GIS after its adaptation, modernization, and development of the block “Snowmelt erosion”, which was performed under the scientific supervision and according to the work of Larionov [38].

The digital elevation model (DEM) used for modeling was developed based on the results of a UAV photogrammetric survey. The spatial resolution of the resulting raster was no more than 5 cm per pixel. The calculation of morphometric parameters and erosion rates was conducted for a grid with cells measuring 10 × 10 m.

### 2.3. Key Catchments

#### 2.3.1. Petrovka Catchment

**Figure 2:**
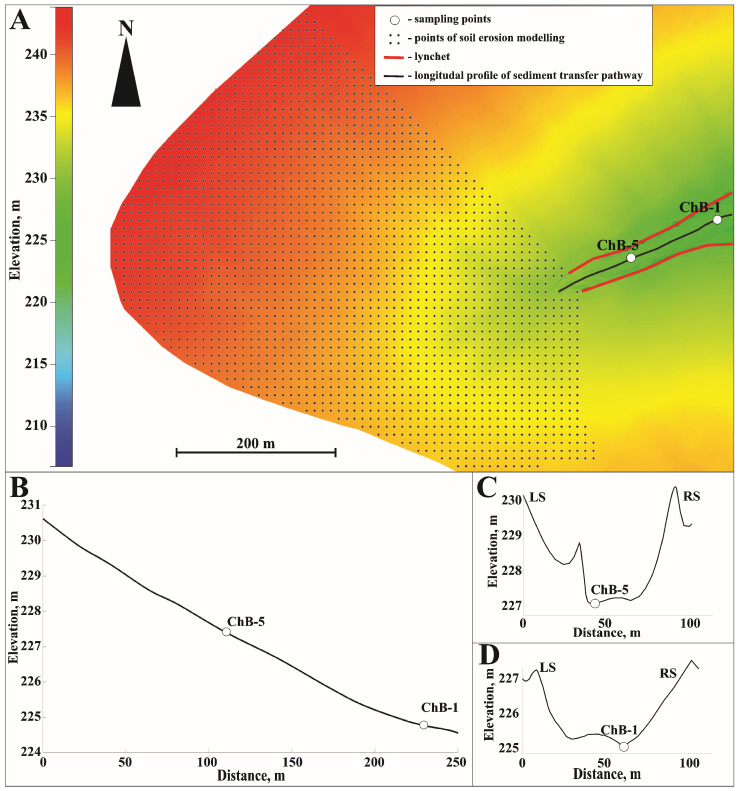
Sampling points within the Petrovka key catchment (**A**), the longitudinal profile of the sediment transport pathway (**B**), and cross-sections of the dry valley at the ChB-5 (**C**) and ChB-1 (**D**) sampling points.

The Petrovka catchment area was studied during two field surveys conducted in 2023 and 2024. During these surveys, the area of arable land that could contribute sediment to the dry valley was approximately 271,200 square meters. This area is located exclusively on slopes at the highest elevations of the catchment area and is characterized by a series of shallow hollows. These hollows act as pathways for runoff and sediment delivery. The terrain of these hollows, however, undergoes significant transformation during tillage. Down by the valley, well-pronounced lynchets are expected to trap slope runoff (Figure 2A). The sampling points were located at the thalweg of a dry valley close to the field edge (ChB-5 in 2024) and at a location where the surface was close to being subhorizontal (ChB-1 in 2023) (Figure 2B–D and Table 1).

#### 2.3.2. Seleznevka Catchment

**Figure 3:**
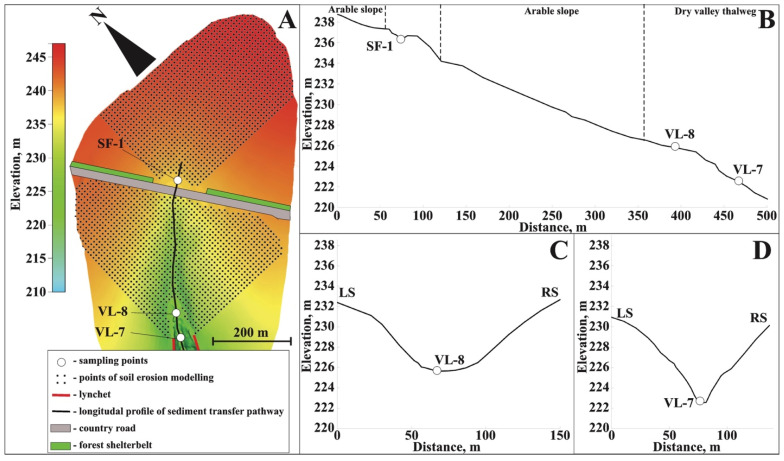
Sampling points within the Seleznevka key catchment (**A**), longitudinal profile of the sediment transport pathway (**B**), and cross-sections of the dry valley at the VL-8 (**C**) and VL-7 (**D**) sampling points.

The upper reaches of the Seleznevka catchment are divided into two parts according to the country road crossing the thalweg of the slope hollow. To the west and east of the intersection with the thalweg, forest shelterbelts have been present since at least the middle of the 20th century and are adjacent to the road upslope. The road embankment and wood form a barrier that traps slope runoff; thus, the only way for sediment to reach the dry valley is by crossing the road at its lowest point in the thalweg (Figure 3A). The depression that existed upstream from the road is now almost filled in, indicating intense accumulation. In the central part of the area, depth incremental sampling was conducted in 2025 (SF-1) (Figure 3B). Since 2015, an irrigation system (presumably the Valley V 8120 model) (Valmont Industries, Inc., Omaha, NE, USA ) has been used in the upper reaches of the area. Although it is unclear exactly how this system is being used, it is likely that this practice has led to increased erosion in the area. Hence, the results of modeling assessments for this part of the catchment should be assumed to be underestimated values.

Downstream from the road, the valley has steep sides and a gentle bottom. Over time, river erosion creates temporary furrows, which are later destroyed by tillage. Freshly accumulated material covering the vegetation was detected during each year of observation. The area upslope from the road can also contribute to the accumulation and sediment yield in the valley. However, quantification of this contribution is challenging. Given the steeper slopes and the larger area of accumulation before the road, it can be assumed that the proportion of sediment supply from the area located below the road is overwhelming.

The hollow slope gradually turns into the vegetated bottom of the dry valley. In 2023, two sampling points were located along the valley’s thalweg, taking into account its morphology. The VL-8 section was set in the upper part, where the valley sides are relatively gentle, and the bottom is wide and has no clear boundaries (Figure 3C). Downstream, the valley narrows significantly, and the steepness of the slope and bottom increases. This implies changes in the sedimentation pattern due to the concentration of potential sediment flow within a compact area. Hence, additional sampling point VL-7 was selected (Figure 3D).

#### 2.3.3. Lapki Catchment

**Figure 4:**
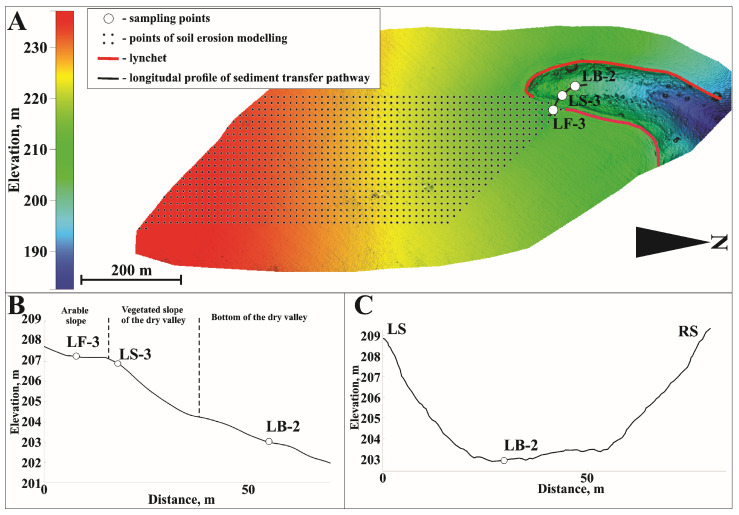
Sampling points within the Lapki key catchment (**A**), longitudinal profile of the sediment transport pathway (**B**), and cross-section of the dry valley at the LB-2 (**C**) sampling point.

The third key catchment area can be considered the most studied area, with numerous studies conducted in this location over the years [39]. The radiocaesium technique has been used to trace the transport pathways and intensity of sediment deposition during the post-Chernobyl period [23,28,40]. Most of the runoff from the slope flows into the valley through a hollow in the center, crossing the lynchet, which has been completely leveled by accumulation [29] (Figure 4A). Sediments were studied within three different geomorphic settings: a filled depression before the lyncher (LF-3), a vegetated slope (LS-3), and the bottom of a dry valley (LB-2) (Figure 4B).

## 3. Results

**Figure 5:**
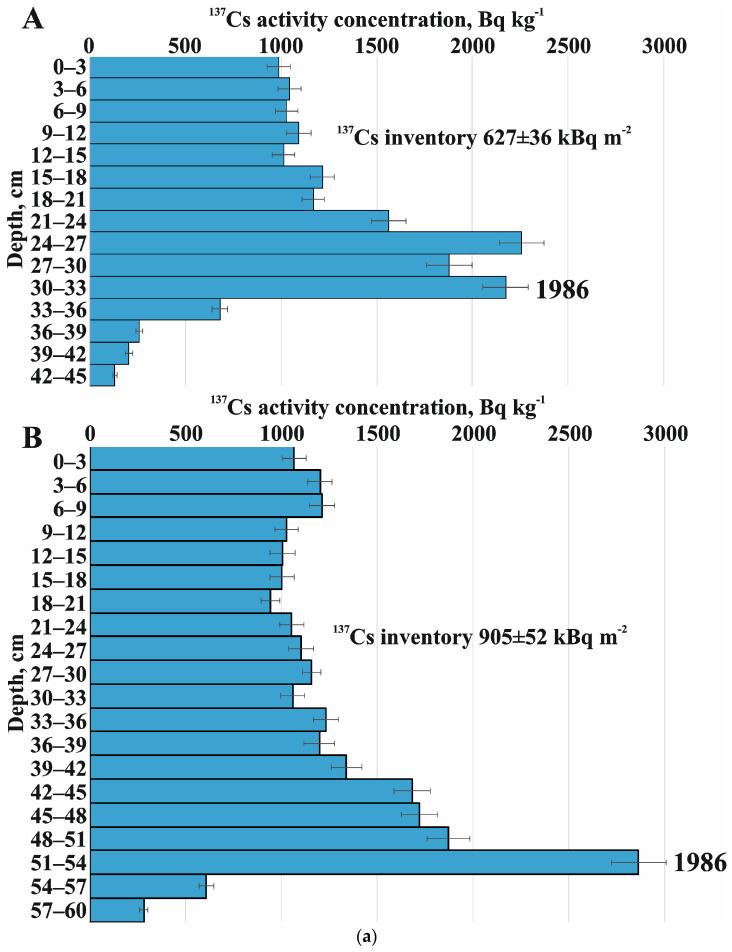

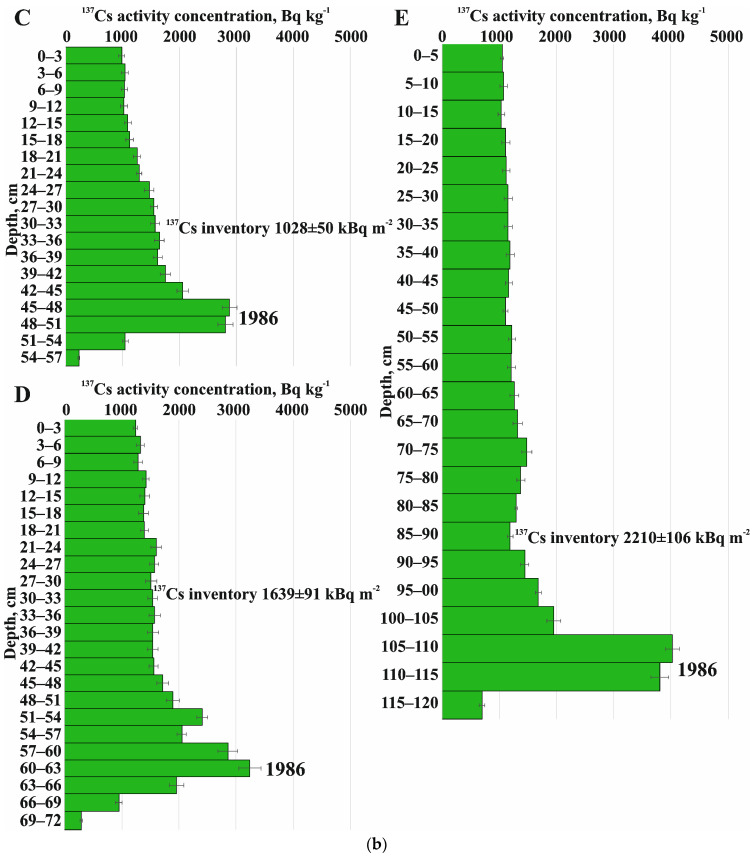

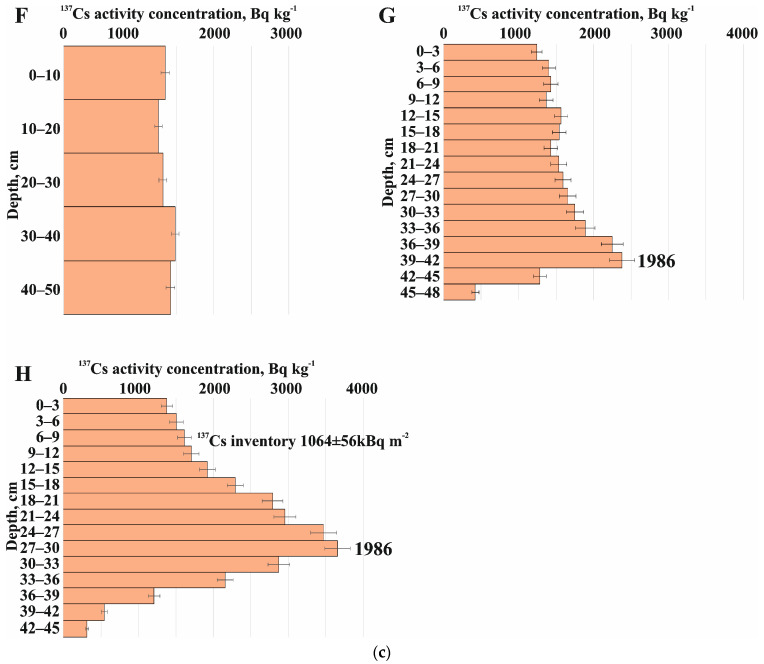
(**a**) Explored depth distributions of the Petrovka catchment: ChB-5 (**A**), ChB-1 (**B**). (**b**) Explored depth distributions of the Seleznevka catchment: SF-1 (**C**), VL-8 (**D**), VL-7 (**E**). (**c**) Explored depth distributions of the Lapki catchment: LF-3 (**F**), LS-3 (**G**), LB-2 (**H**).

The obtained ^137^Cs depth distributions are shown in Figure 5, and the soil erosion modeling is shown in Supplementary Figure S1. Based on the observed data, it can be tentatively concluded that the sampling depth used was sufficient to determine the overall Chernobyl-derived ^137^Cs inventory, with possible exceptions for LF-3 and LS-3. In all cases, the assessed values of the ^137^Cs inventory are much greater than the initial fallout values of 1986 (Figure 1C): Chb-5 (Figure 5A)—627 ± 36 kBq m^−2^; ChB-1 (Figure 5B)—905 ± 52 kBq m^−2^; SF-1 (Figure 5C)—1028 ± 50 kBq m^−2^; VL-8 (Figure 5D)—1639 ± 91 kBq m^−2^; VL-7 (Figure 5E)—2210 ± 106 kBq m^−2^; and LB-2 (Figure 5H)—1064 ± 56 kBq m^−2^. Therefore, the results indicate intensive post-Chernobyl accumulation, and the selected sites can be used as an environmental archive.

The depth distribution of ^137^Cs can be recognized as a record of variations in the activity concentration of the sediment load from a particular source if the following constraints are considered:

1. The mixing of material before it leaves the source is adequate. This assumption can be indirectly supported by three facts. The first is the constant mixing of the material during tillage, which occurs in different directions from year to year. Second, given the microtopography of the explored area, slope runoff must be concentrated to overcome the lower boundary of cultivated fields [28]. This, in turn, leads to intensive mixing within the compact areas of the slope hollows and a limited number of sediment delivery paths [29]. The latter suggests that the desired samples may be collected at a few points near the lower boundary of the cultivated field.
2. There are no significant changes in the sediment during transportation due to sorting and selective conveyance. The sorting of material by grain size is a common process that may occur during the delivery of the material. This process may seriously affect the concentration and inventory of radionuclides by altering the proportion of particles that have a greater or lesser ability to bind to radionuclides [41]. Clearly, the shorter the transportation distance, the lower the probability of the material being sorted. Hence, the proximity of the sampling point to the field is an important factor. However, examinations of the mechanical composition in the Lapki catchment area revealed no significant differences between eroded soil and deposited sediments [28,39,42].
3. The sediment trap efficiency at the selected depositional sites should remain stable over the observation period to provide a homogeneous reflection of concentration variations in the depth distribution. This requirement is nearly impossible to fulfill because numerous factors influence the accumulation process, which may change over time. Even seemingly stable factors such as topography are expected to be altered as a result of the accumulation and uplift of the surface. To some extent, the only way to manage this issue is to collect and compare samples from different locations based on geomorphic features.
4. After sediments are deposited, the depth distribution of ^137^Cs should not be altered to preserve the natural record of radionuclide migration. The main mechanisms of disturbance are the leaching and migration of radionuclides through the soil profile, bioturbation, and erosion. Repeated depth incremental sampling conducted in the Lapki catchment in 1997 and 2024 revealed that, after 27 years, the distribution smoothed [39]. Despite the high pH values of the chernozem and chernozem-like soils, the leaching of ^137^Cs was minor, and radionuclides migrated mostly in a bound form. This circumstance indicates that the interpretation of the obtained data may be biased, resulting in a slight decrease in the contrast of the original distribution. However, younger sediments are less affected than older sediments. The bioturbation process can be considered via detailed observation of the soil section before sampling. The obvious tracks of biota influence, such as mole holes or roots, serve as indicators for selecting the correct position for sampling. In addition, the presence of subhorizontal textures, which are typical of the sequential accumulation of sediments, can be used as evidence of a lack of mixing. The erosion of deposited sediments occurs mainly in the form of a linear incision into the bottom surface and is usually well defined morphologically. The most intense form of erosion is the incision of bottom gullies, which can result in the complete displacement of contaminated layers. Consequently, thoroughly examining the morphological features of the deposition site after a fallout event before determining the location of the sampling site is essential.
5. Finally, the size of the samples during the depth incremental procedure is set arbitrarily (3–5 cm thick); therefore, the averaging of activity concentrations within selected depth intervals may affect the representation of the true distribution.

To summarize, it should be noted that ^137^Cs depth distributions do not provide a perfect record, such as in any other environmental archive. The data obtained are always a compromise between the desire for the most representative information and actual natural conditions and possibilities, but careful analysis is needed.

## 4. Discussion

### 4.1. Assumptions and Expectations

Field experiments conducted immediately after the incident indicate that the typical time for ^137^Cs to become fixed in most soils is on the order of dozens of days [43]. This, in turn, implies that there was a very brief period of time during which radionuclides migrated in their unbound state. After that, the ^137^Cs inventory was altered, mainly by the redistribution of contaminated sediments and radioactive decay. The latter is an independent process, and its contribution is equal in all cases. This can be considered via a decay correction calculation.

The concentration of ^137^Cs in eroded material depends on the radionuclide depth distribution in the soil profile. The main factor controlling the concentration was mixing during plowing. This immediately resulted in a decrease in concentration of up to 10 times and was believed to be an effective countermeasure [44,45]. However, plowing in the Lokna River basin at the beginning of May 1986 was likely to have been completed, and no further manipulations were carried out before harvesting at the end of the summer. Moreover, the use of deep plowing, including turnover, shortly after fallout is highly unlikely because the first official contamination map was published in 1989 [46]. The standard method of soil cultivation in the USSR involves plowing the land to a depth of 25–30 cm every five years. After fallout, it took several years to mix and distribute ^137^Cs evenly within the upper 30 cm, and this time gap could vary from field to field. Thus, the significant decrease in activity concentration in topsoil resulting from plowing was not instantaneously and uniformly distributed across the entire catchment area.

After the radionuclides are mixed with the arable horizon, their concentration in the surface layer, which is potentially susceptible to erosion, is determined by three factors: vertical migration along the soil profile, erosion, and the deposition of soil matter that has been transferred from the upper slope. The first factor is expected to be negligible because of the nonacidic chemical conditions and the high proportion of clay particles in the soil. Typically, the depth distribution of Chernobyl-derived ^137^Cs under undisturbed conditions can be described by an exponential decrease in concentration with depth [47]. Erosion leads to a decrease in the soil concentration because the replacement of upper soil by the same depth of plowing leads to the involvement of cleaner material from deeper horizons. This mechanism of attenuation could have a significant effect if deep plowing is repeated. Otherwise, changes in concentration would only occur after the thickness of the eroded layer is large enough to allow for the uplift of the underlying soil during less intensive plowing. During intraslope accumulation, especially at the foot of slopes or in local depressions, the thickness of the layer containing a high concentration of radionuclides increases. However, these sites can potentially erode, and cesium-rich sediments may be reintroduced into the sediment load. This phenomenon is unlikely to disrupt the general downward trend, but it may smooth it out.

Based on the information provided, the record of ^137^Cs transfer from a specific source is expected to consist of two main parts: The first part is a sharp increase (“peak”), which could be due to both radioactive fallout and the migration of radionuclides immediately afterward. The second part is a generally decreasing trend (“drop”), with fluctuations caused by local features of contamination and redistribution of sediments.

### 4.2. Interpretation of the ^137^Cs Depth Distribution

In the study area, the level of ^137^Cs deposition did not exceed 7.4 kBq·m^−2^ before the accident [27]. After that, it increased by almost two orders of magnitude (see Figure 1C). Therefore, a sharp increase in radionuclide activity concentration is expected in the sediment due to atmospheric deposition in 1986, and this increase may be used for absolute geochronology. However, the direct link between the highest activity concentrations and fallout may be disputed if the deposition site is less contaminated than the area that provided the material for subsequent sedimentation.

At all observed points, a “peak” was detected, and the moment in 1986 was determined. The only exception was LF-3 (Figure 5F). In this case, the soil had been previously subjected to tillage, and the sampling depth was not deep enough to capture all the contaminated layers. There are two possible approaches for interpretation: (i) assume that the sample with the highest value is the result of fallout, or (ii) find a sample that corresponds to the peak of the exponential distribution. Under undisturbed conditions, without erosion or accumulation, these two samples are expected to be the same.

The first approach is simpler and uses clear and concise criteria. However, as seen in the example of the Chb-5 section (Figure 5A) in the Petrovka catchment, there are two separate samples with comparably high activity concentrations. Following the first approach and assuming that the highest value at a depth of 27–30 cm corresponds to fallout, the explanation for the distribution down the profile becomes unclear. A similar situation can be observed for LF-1 at 45–48 and 48–51 cm (Figure 5C) and for VL-7 at 105–110 and 110–115 cm (Figure 5D). A similar situation can be observed for LF-1 at 45–48 cm and 48–51 cm (Figure 5C) and for VL-7 at 105–110 cm and 110–115 cm (Figure 5D). Two explanations exist for the presence of these pairs of samples with similar activity concentrations: First, during sampling, materials from different depth intervals may have been mixed together. In the upper sample, radionuclides from the 1986 fallout may have been mixed with older, deeper material, whereas in the second sample, they may have been mixed with sediment accumulated after the fallout. This may result in an even curve in this interval. The second factor is the high activity concentration in sediments that accumulated shortly after 1986, which was equal to or even greater than that from the direct deposition of ^137^Cs. Both factors may contribute simultaneously.

Although a visual analysis of the graph seems to be an appropriate approach for identifying the exponential part formed by direct fallout, this method is somewhat qualitative. The exponential shape of the ^137^Cs depth distribution in undisturbed soil is widely recognized in radiocaesium studies [47,48,49]. Using this method, it can be concluded that there was an intense supply of particulate radionuclides, which may complicate the dating process.

Another piece of evidence suggests that the lateral transport of high-intensity radionuclides is recorded in depth distributions and is closely dependent on geomorphic conditions. However, it should be noted that our assumptions are based on modern topography, which differs from that of 1986 and therefore may contain bias. The “peak” has a higher activity concentration when the morphometric conditions suggest a higher trap efficiency: the lower slope angle (Table 1) in the case of the Petrovka (Figure 5A,B) and Lapki catchments (Figure 5G, H) and the concentration of the sediment load due to the narrowing of the dry valley bottom (Figure 3C,D) in the Seleznevka catchment (See Figure 5D,E). The delivery distance can also be a significant factor, as it can lead to a loss of water flow energy and subsequent sediment accumulation. In other words, the more likely it is that the sediments mobilized by slope runoff will pass through the studied point without accumulating, the less sharply the “peak” will look. One point that can be recognized is the obvious fact that the proximity of ^137^Cs inventories from various origins in a single soil sample greatly complicates the interpretation and reliability of conclusions about the migration of radionuclides in the early years after an accident.

As we move upward, we observe a general decrease in activity for all the studied distributions. In general, two stages can be distinguished: an intense decline followed by a relatively stable level. However, the picture may differ significantly in each case. In the soil samples from Petrovka, the upper layers show an even distribution of ^137^Cs (Figure 5A), and there are also moderate fluctuations (Figure 5B). This pattern is generally consistent with expectations, as it indicates that, over the past few years, there has not been a significant decrease in activity concentration since mixing in the plowing layer, which is in line with the lowest estimated erosion rates among the other sites.

The rate of sedimentation is higher due to the geomorphic conditions, such as the larger catchment area and higher erosion rates. The latter are believed to be underestimated because of the use of an irrigation system upstream from the SF-1 section. Unfortunately, the contribution of human-made slope runoff cannot be adequately assessed based on the given data. In the SF-1 section (Figure 5C), there is a nearly constant downward trend, whereas in the VL-8 and VL-7 sections, the values seem to remain stable over a longer period of time (Figure 5D,E). This difference is consistent with the impact of erosion caused by the irrigation system upstream of SF-1. Irrigation may also affect VL-8 and VL-7, but its contribution is likely to be significantly reduced by sedimentation upstream of the road. However, the mean erosion rates assessed via the model should still be considered underestimated.

Sections from the Lapki catchment show that accumulation is strongly related to geomorphic features. The activity concentration changes over time and can be used as an indicator of sediment age: younger sediment has a lower activity concentration [23]. Based on this assumption, recently eroded material with activity concentrations less than 1500 Bq·kg^−2^ is intensively redeposited at the foot of the slope (Figure 5F) and on the dry valley side (Figure 5G). Moreover, the bottom material appears to be more active, indicating an older age (Figure 5H). This pattern can be explained by a decrease in geomorphic connectivity after the Chernobyl accident due to climate change. Initially, the energy of slope runoff was sufficient to transport sediment more effectively down the valley to the bottom, where it accumulated. However, owing to the less intense water supply from snowmelt in spring in the XXI century [50], sediment is now trapped mostly on valley slopes.

The total decrease in concentration during the post-Chernobyl period, as determined based on sediments in the explored catchments, varied significantly. At the Petrovka site, the decrease was less than twofold, ranging from 930.5 to 1258 Bq/kg in ChB-1. A more significant reduction was observed in the LB-2 section in the Lapki catchment, where the values decreased by more than two times, with a variation from 3 469.5 to 1 380.3 Bq kg-1. The sections in Seleznevka also showed intense post-fallout changes in activity levels: VL-8—2.3 times (from 2858.7 Bq·kg^−1^ to 1243 Bq·kg^−1^); SF-1—2.91 times (from 2883.8 Bq·kg^−1^ to 991.5 Bq·kg^−1^); and VL-7—3.83 times (from 4031 Bq·kg^−1^ to 1053 Bq·kg^−1^) (see Table 2). Obviously, even in closely located areas with the same average rate of erosion upstream, the ratio of activity concentration immediately after 1986 compared to the current value may vary. This difference may indicate that topography can influence sediment transport and sediment trap efficiency in a particular geomorphic unit, especially during short-term, high-magnitude erosion events.

The estimation of activity concentration changes can be subject to two major challenges: The first challenge is differentiating between radioactive fallout and inventories of ^137^Cs that have accumulated in sediments, as previously discussed. This can result in both over- and under-estimating the initial activity concentration. The second challenge is that the dominantly downward vertical transfer of radionuclides through the soil profile has affected older layers to a greater degree than younger layers. This is more likely to lead to an underestimation of both the initial activity concentration and the total decrease. Although there are uncertainties in the analysis of the depth distribution, it can be concluded that, in the observed cases, the long-term variations in the concentration of particulate ^137^Cs are correlated with soil erosion.

### 4.3. Long-Term Modeling of Particulate ^137^Cs Migration

As erosion is a major factor in the transport of particulate radionuclides, it is essential to determine how to evaluate these processes appropriately. The simplest way to collect data is through direct observation during an experiment. The first experiment was conducted between 1995 and 1996 and used a 20 m × 20 m runoff plot in Ukraine’s Chernobyl-affected zone with rain simulation. Even with enforced runoff, the decrease in the ^137^Cs inventory was only 0.07%. Therefore, soil erosion under these conditions seems to be the third most significant factor in reducing radioactivity after radioactive decay and the removal of soil clumps on the roots of vegetation during weeding and harvesting [51]. Owing to the laborious nature of the procedure and occasional data losses [52], such studies are not numerous [53,54,55,56]. After the accident at the Fukushima-1 nuclear power plant, direct observations became relevant again [56,57,58]. On a USLE plot (5 m × 22.1 m), which had been left unplanted for a year due to continuous weeding, the loss of radioactive cesium through water erosion was approximately 10% each year. In contrast, in vegetated farm plots, erosion losses are much lower, well below 0.01% per year [59]. Even though experimental studies have great value, they remain very limited in number, which encourages the use of mathematical modeling.

When studying the behavior of Chernobyl-derived cesium in rivers, the main focus of empirically based modeling efforts has been on assessing the transport of radionuclides in dissolved and particulate forms, as well as the exchange between them [60,61,62,63]. The importance of accelerated erosion in the migration of radiocaesium on a large scale within river systems has been highlighted for both Chernobyl- and Fukushima-affected areas [64,65,66,67,68]. The development of physically based models involves considering the sediment supply and its distribution [62,69,70,71,72,73]. This study suggests that investigations of sedimentary environmental archives could provide valuable data for verifying and improving such models.

## 5. Conclusions

1. Depth incremental studies of accumulated products of accelerated erosion can be instrumental in understanding long-term trends in the transport of radionuclides in the environment. However, several factors may affect the depth distribution of ^137^Cs; thus, they should be carefully considered during planning, sampling, and interpretation. The consideration of geomorphic conditions at the sampling site is critical, as topography is the main factor that controls spatial heterogeneity in sediment displacement and accumulation processes.
2. Soil tillage including deep plowing is the dominant factor in determining the decrease in the activity concentration of ^137^Cs in sediment runoff from agricultural slopes. However, owing to the lack of immediate remediation measures after the Chernobyl accident in areas distant from the power plant, the ^137^Cs content in the top layer of soil subjected to erosion is expected to remain relatively high. This led to intensive migration of particulate radionuclides shortly after fallout. During ordinary plowing, the downward trend became less significant, and further changes in activity concentration were primarily influenced by erosion.
3. The thickness of the eroded upper soil has become large enough to allow the involvement of soil material from deeper layers during ordinary plowing. This may lead to a subsequent reduction in the ^137^Cs activity concentration. Future forecasts should be based on an understanding of accelerated erosion and its contributing factors, including climatic change and land use conversion.

## Figures and Tables

**Table 1:** Morphometric parameters of the sampling sites.

Sampling Point	Slope Angle in Sampling Point *	Distance from the Cultivated Field, m	Upslope Catchment Area, 10^3^ m^2^
ChB-5	1.5	105	271.2
ChB-1	0.7	223	
SF-1	0.4	20	133.6
VL-8	0.9	94	266.8 **
VL-7	2.6	193	
LF-3	0.5	0	102.2
LS-3	6.9	8	
LB-2	4.2	55	

Note: * angle of modern surface; ** including the area upstream of the road.

**Table 2:** Changes in the ^137^Cs activity concentration in the sediments accumulated over the post-Chernobyl period at the sampling points.

Section	Mean Rate of Erosion Upslope	^137^Cs Activity Concentration, Bq kg^−1^			>1986/ 2022–2025
		Shortly After Fallout (>1986)	Surface Layer (<2022–2025)	Range	
ChB-5	3.6	1878.9	987.9	1268.4	1.9
ChB-1	3.6	1871.7	1065.7	930.5	1.76
SF-1	>2.6	2883.8	991.5	1892.3	2.91
VL-8	>7.6	2858.7	1243.3	1615.4	2.3
VL-7	>7.6	4031	1053	2978	3.83
LS-3	15.5	2246.1	1241.8	1004.3	1.81
LB-2	15.5	3469.5	1380.3	2089.2	2.51

## Data Availability

The original contributions presented in this study are included in the article and Supplementary Materials. Further inquiries can be directed to the corresponding author.

## Supplementary Materials

The following supporting information can be downloaded at: https://www.mdpi.com/article/10.3390/toxics14040344/s1, Figure S1: Results of erosion modeling for Petrovka (A), Seleznevka (B), and Lapki (C) catchments.
