# Supplementary figures and images for: The Long-Term Dynamics of the Particulate 137Cs Supply from Eroded Arable Slopes During the Post-Chernobyl Period
Source: Toxics. 2026 Apr 19;14(4):344. doi: 10.3390/toxics14040344 (PMC13120182; doi:10.3390/toxics14040344)

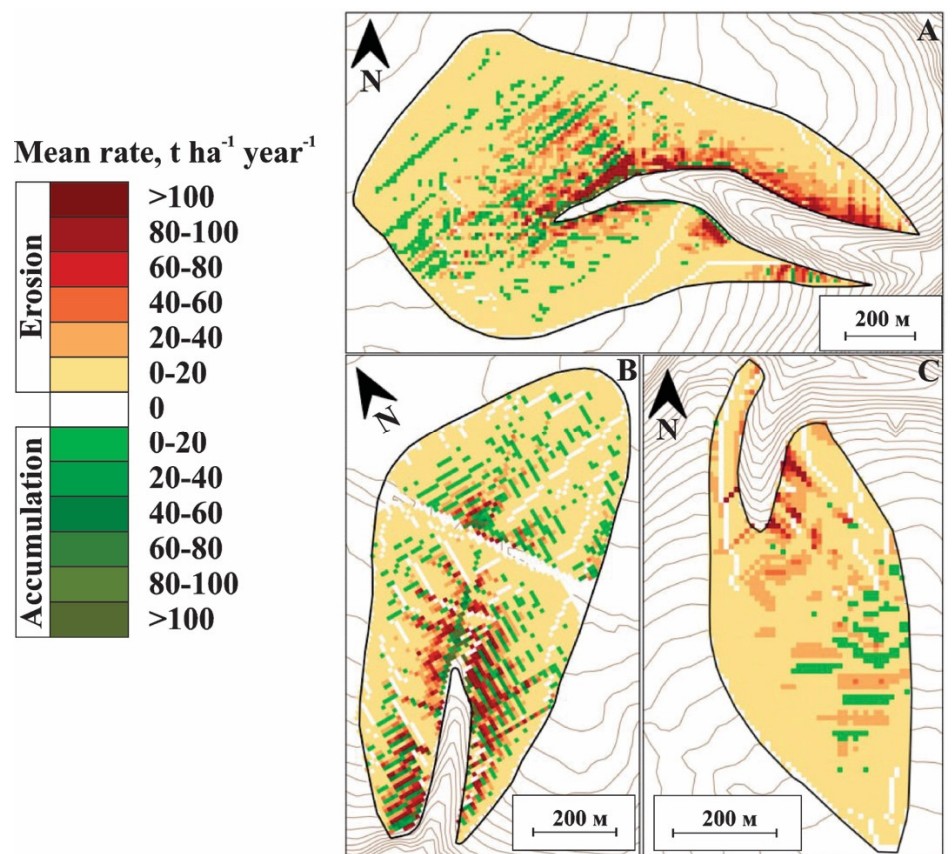

**Figure SM-1.** Results of erosion modelling for Petrovka (A), Seleznevka (B) and Lapki (C) catchments.

Supplement: Supplementary file 1 [file toxics-14-00344-s001.zip › toxics-4221733-supplementary.pdf]
